# Screen time and associated risks in children and adolescents with autism spectrum disorders during a discrete COVID-19 lockdown period

**DOI:** 10.3389/fpsyt.2022.1026191

**Published:** 2022-12-01

**Authors:** Mathilde Berard, Marianne Peries, Julie Loubersac, Marie-Christine Picot, Jonathan Y. Bernard, Kerim Munir, Amaria Baghdadli

**Affiliations:** ^1^Centre de Ressource Autisme Languedoc-Roussillon, CHU Montpellier, Montpellier, France; ^2^Centre d'Excellence sur l'Autisme et les Troubles Neuro-développementaux, CHU Montpellier, Montpellier, France; ^3^Université Paris-Saclay, UVSQ, Inserm, CESP, Team DevPsy, Villejuif, France; ^4^Clinical Research and Epidemiology Unit, Department of Medical Information, University Hospital, CHU Montpellier, Montpellier, France; ^5^Université Paris Cité, Inserm, INRAE, Centre de Recherche en Épidémiologie et Statistiques, Paris, France; ^6^Singapore Institute for Clinical Sciences (SICS), Agency for Science, Technology and Research (A*STAR), Singapore, Singapore; ^7^Developmental Medicine Center, Boston Children's Hospital, Harvard Medical School, Boston, MA, United States; ^8^Faculté de Médecine, Université de Montpellier, Montpellier, France

**Keywords:** autism spectrum disorder, COVID-19, lockdown, screen time, children, adolescents

## Abstract

**Background:**

The COVID-19 pandemic may affect the screen time of children and adolescents with Autism Spectrum Disorders (ASD). This study aimed to examine the screen time of children and adolescents with ASD during a discrete lockdown period in France and identify risk factors for excessive screen time.

**Methods:**

The study sample consisted of 249 ASD subjects, 3–17 years of age, enrolled in the ELENA cohort. Information about the screen time was collected using the COVID-19 questionnaire specially created for this study. The clinical, socio-demographic and familial characteristics were collected from the last ELENA follow-up visit.

**Results:**

More than one third of subjects exceeded recommended levels of screen time and almost half of parents reported that their child spent more time using screen since COVID-19 pandemic beginning. Excessive screen time was significantly related to age with higher screen time in adolescents. Risk factors for excessive screen time were high withdrawn behaviors and low socioeconomic status for children, and older age and male gender for adolescents.

**Conclusion:**

These results imply to adapt the recommendations already formulated in general population concerning the good use of screens in youth with ASD. Specific recommendations and suitable guidance are needed to help children and adolescents with ASD and their parents implement the more optimal use of screen time activities for educational, therapeutic and social goals.

**Trial registration number:**

NCT02625116.

## Introduction

The use of television and mobile devices by children and adolescents with autism spectrum disorders (ASD) occupies a central place in their everyday lives. Current measure of screen time is the total duration (usually in hours/day) of time spent viewing programs, or playing video games, on television, a computer, or a mobile device. Other consistent finding is that, over the ensuing decades, the screen time among children and adolescents has been increasing. To date, studies of screen time of children and adolescents in the general population pointed to poorer health outcomes in terms of sedentary health risks, behavioral strengths and difficulties scores, prosocial behaviors (1, 2), psychological wellbeing, quality of life (3), sleep (4), as well as depressive (3, 5), and anxiety disorders (6). It has also been suggested that excessive screen time can lead to worsening of autism-like symptoms (7–10), attention deficit hyperactivity disorder (ADHD) (11, 12), dyslexia (13), as well as language, cognitive, and motor acquisition delays (14, 15).

To date, the indicators of risk for excessive screen time in children and adolescents in the general population have included male gender, older age, urban residence, insufficient home living and play space, lower parental education, lower household income, and inconsistent parenting practice in terms of ability to control screen time behaviors (16–20).

Given the concerns about the negative impact of excessive screen time of children and adolescents, a number of international guidelines have been proposed (21–24). The guidelines consistently recommend for children and adolescents not to exceed an hour of screen time daily for ages 2–5 years, and 2 h daily for ages older than 5 years. Surprisingly, studies have consistently found that at least half the children in the general population exceed these limits (19, 25, 26) increasing with age (25, 27).

Although currently there are no specific recommendations on acceptable screen time in children and adolescents with ASD, studies consistently show that they spend more screen time compared to their typically developing peers (28–32) and may therefore be at greater risk of becoming dependent (33–35), or attracted to video gaming that may correspond to their solitary and repetitive social patterns of interaction (36). Number of studies have also pointed to unfavorable effects of excessive screen time in children and adolescents with ASD that included enhanced sedentary behavior (29, 31, 32), ADHD symptoms (33), reduced mother-child reciprocal interaction (30) and sleep problems (37–39). The National Survey of Children's Health (26) involving a representative sample of U.S. children, age 6–17 years, showed that although more than half of children with ASD were high users (more than 2 h/day), nevertheless compared to non-ASD children, they had similar amounts of screen time. Montes (26) has further cautioned against assumption of increased risk of excessive screen time among children and adolescents with ASD especially given the utility of visual electronic devices in ASD as communication and teaching aids.

A recent study examining the impact of the COVID-19 pandemic on health behaviors among adolescents with ASD reported significant increase in screen times in terms of both weekday (3.7 h/week) and weekend (5.9 h/week) use (39). Others studies also emphasized the negative effect of the COVID-19 lockdown on youth physical activity and recreational opportunities during the lockdown (40, 41). The stay-home orders during the COVID-19 pandemic have led to doubling or tripling of screen time among children and adolescents with ASD (16, 19, 39–46).

The purpose of the current study was to examine and describe screen time in a sample of children and adolescents with validated ASD diagnoses enrolled in the ELENA cohort study in France during a discrete lockdown period, and to identify clinical and socio-demographic risk factors for excessive screen time.

We first hypothesized that screen time of children with ASD will increase during the lockdown period, as observed by previous studies. Moreover, based on prior studies, we expect to identify several risk factors for excessive screen time in these children, both clinical (severe autistic symptoms, low intellectual and adaptive functioning and behavioral issues) and socio-demographic (low parental educational levels and socioeconomic status).

## Methods

### Study design

The present study is a cross-sectional survey of parent-informants of children and adolescents with ASD enrolled in the ELENA regional cohort in France carried out between November 5 and December 18, 2020 corresponding to the second COVID-19 lockdown in France. The participants were recruited from the ELENA cohort, an ongoing, prospective, and multicenter study of developmental trajectories in ASD. The subjects followed in the study were 2–16 years at inclusion (V0), all with confirmed ASD according to DSM5 criteria and multidimensional assessments including ADOS, ADI, and psychological assessments. Inclusions in ELENA were carried out over a period from 2013 to 2019. Thereafter, all children are followed for 72 months with standardized clinical data collection times at 36 (V1) and 72 months (V2) after inclusion. The clinical data collected at these times are similar. Complete details about the ELENA protocol have been published elsewhere (47).

### Participants

For the present study, the participants consisted of children with a confirmed diagnosis of ASD and fulfilled the following inclusion criteria: (1) active follow-up in the ELENA cohort; (2) aged 3–17 years; (3) completed COVID-19 questionnaire; and (4) living full-time or shared custodial arrangement with the responding parent.

The 249 children and adolescents were mainly boys (80.3%) and their mean age was 9.1 years (SD = 3.8). Fifty height percent of children had a middle or high parental socioeconomic status (SES). Over half of the mothers (62.2%) and the fathers (53%) had a college or university education. Children involved in this study were younger and had a higher estimate intellectual level and VABS-II scores for communication and daily living skills then the other children included in the ELENA cohort (Supplementary Table 1).

### Procedure

For the present study, parents were invited by a letter to complete an online questionnaire related to the specified lockdown period via the ELENA cohort database electronic system. A reminder was sent to parents 2 weeks later by e-mail or telephone. Only one questionnaire was completed per child by corresponding parent. As the COVID study was carried out in addition to the ELENA follow-up, we did not automatically collect the motives for declining to participate in this survey, but the few families who did cite a lack of time. Signed informed consent has been obtained from all participating families included in the ELENA cohort. This study was approved by the Internal Review Board of the University Hospital of Montpellier and was conducted according to the recommendations of the Declaration of Helsinki.

### Measures

#### COVID-19 questionnaire

The COVID-19 questionnaire included: (i) child characteristics, education and leisure activities, specialized care (i.e., speech therapy, occupational therapy, psychosocial intervention; social skills training), sleep (within the last 15 days), social home environment and relationships; and (ii) child screen time from the inclusion of the pandemic in March 2020: average screen time (hours/day) assessed on a 5-point Likert response scale (none; <1 h/day; 1–2 h/day; 2–4 h/day; >4 h/ day), change in screen time since the COVID-19 lockdown (November 5–December 18, 2020), assessed as increased, decreased, same as before, or not applicable (i.e., “my child does not spend any time on screens”).

#### Clinical characteristics

The clinical characteristics of subjects have been previously assessed by licensed study psychologists using standardized tools within the ELENA Cohort. The child and adolescent adaptive skills were assessed with the Vineland Adaptive Behavior Scales, Second Edition (VABS-II) (48). Autism symptom severity was measured using the calibrated severity score (CSS) of the Autism Diagnostic Observation Schedule-2 (ADOS-2) (49, 50). A best estimate intellectual functioning was calculated using standardized and validated instruments [Brunet-Lézine R (51); BECS (52); PEP-3 (53); WPPSI-IV (54); WISC-V (55); WAIS-IV (56); K-ABC (57)] based on child age and level, according to the approach of Howlin et al. (58). Psychiatric comorbidities were assessed using the Child Behavior Checklist [CBCL; (59)], a norm-referenced measure assessing emotional and behavioral disorders in children. A CBCL score <65 corresponds to a normal range and a score greater or equal to 65 corresponds to a borderline or clinical range. Children and adolescents responses to sensory stimuli were assessed by the Sensory Profile (SP), a parent-reported 125-items questionnaire (60) for which a total score (SP total score) was calculated from 36 items (61), lower scores indicating greater SP difficulties. The tools and measures have been described in detail in the ELENA protocol (47). The clinical characteristics used in this paper were collected at the last ELENA follow-up visit closest to the beginning of the containment with a mean delay of 12 months between these times (standard deviations ± 10.7).

#### Socio-demographic and familial characteristics

The socio-demographic and familial characteristics included: sibship size, household composition, parental ages, educational levels and socioeconomic status (SES) studied as a composite variable based on the mother and/or the father's professional background. When the parents were living together, the highest SES in the household was taken into consideration; when separated, the SES of parent with whom the child lived most of the time was taken into consideration. Only current occupational professional activities were considered; if a parent did not work at the time of the study, her/his SES was not taken into account. Parental SES was scored as high (business owners, professionals, executives), middle (farmers, supervisors, skilled craftsmen), or low (farm workers, laborers, service employees, and unemployed). Middle and high SES classes were grouped to increase the power of data. All data about socio-demographic and familial characteristics used in this paper were extracted from the last ELENA follow-up visit.

### Statistical analysis

The outcome variable was screen time of children and adolescents during the specified COVID-19 lockdown period, defined as greater than recommended (21, 23) (<1 h/day for 2–5-year-old group, and <2 h/day for >5-year-old group). The following potential explanatory variables were considered for the analysis: (i) data from ELENA follow-up: for the child (gender and clinical characteristics: CSS, VABS-II, CBCL and SP scores, best estimate intellectual functioning), and the parents (number of siblings, parental SES and educational level); and (ii) data collected during lockdown: for the child (age, education, leisure activities, weekly social relationships with peers and family and continuation of specialized care) and the parents (age, household composition and perception of their child's sleep).

Descriptive analysis was performed according three age subgroups identified according to school levels (preschool, elementary and beyond than middle school, respectively): <6 years old, 6–11 years old and ≥12 years old. Means with ± standard deviations (SDs) were reported for continuous variables. Categorical variables were described into frequency and percentage. Pearson chi-square or Fisher exact tests and ANOVA tests were used to compare sub-groups characteristics. Pairwise comparisons were performed using the Bonferroni *post-hoc* test. Chi square test was used to compare the excessive screen time between groups.

The risk factors analysis was performed for the subgroups: children (<12 years) and adolescents (≥12 years). The groups <6 years, and 6–11 years, were pooled into a group < 12 years, because of (1) the homogeneous prevalence of screen time for the age subgroups <6 years and 6–11 years and to address (2) the limited sample size of the age subgroup <6 years.

The association between potential explanatory factors and screen time during the lockdown was studied using Pearson chi-square or Fisher exact tests for the categorical variables and T-Test or Kruskal-Wallis tests for continuous variables. The following effect sizes, Cramer's V, Cohen's d and eta^2^, were calculated as appropriate (62).

For the multivariable logistic regression models of excessive screen time risk factors, variables with a *p* <0.20 according to the univariate analysis were included in the model and the model that minimized the Akaike Information Criterion (AIC) was selected using a backward selection. The multivariable model for the <12 years group was adjusted with the delay between the date of the last CBCL and the filling time of the COVID-19 questionnaire. The adjusted odds ratios (aORs) with 95% confidence intervals (CIs) are presented. The goodness-of-fit of the models was assessed using the Hosmer and Lemeshow test. A sensitivity analysis was performed using *chained equation missing data method* to impute the missing explanatory variables (100 imputation datasets), assuming that the data were missing at random. All statistical tests were considered significant for *p* < 0.05. Statistical analyses were performed using SAS Enterprise Guide V7.13 (SAS Institute Inc., Cary, NC, USA).

## Results

### Population

The sample study included 249 children and adolescents who were mainly boys (80.3%). The caregivers who filled the COVID-19 questionnaire were mothers for 202 participants (81.2%), fathers for 20 of them (8%) and both parents combined for 27 of them (10.8%). The means ADOS-2 CSS was 7.37 (*SD* = 1.8) and the mean estimate intellectual functioning was 79.1 (*SD* = 30.2). The mean VABS-II scores were 73.3 (*SD* = 17.4) for communication, 73.0 (*SD* = 15.7) for daily living skills and 69.1 (*SD* = 14.3) for socialization. The three age groups considered for this study: <6 years (*n* = 40, 16.1%); 6–11 years (*n* = 143, 57.4%); and ≥12 years (*n* = 66, 26.5%) were comparable on CSS and VABS-II scores but significantly different on intellectual level (*p* < 0.001, eta^2^ = 0.17), that increased with age. Most children were in school (*N* = 225, 90.4%) and had no leisure activities (*n* = 169, 67.9%). Specialized care was maintained for most children and adolescents (*n* = 227, 91.2%) but it was significantly more frequent in 6–11 years than ≥12 years subgroup (94.4 and 83.3%, respectively; *p* = 0.01). More details are presented in Table 1.

**Table 1:** Children, family, and socio-demographic characteristics.

	**<6 years**	**6–11 years**	**≥12 years**	**p-value**	***Post hoc*** **-test**	**Effect size**
	**(G1)**	**(G2)**	**(G3)**			
	***N*** **= 40**	***N*** **= 143**	***N*** **= 66**			
**Children and adolescents' characteristics**						
**Gender**						
Boys	33 (82.5)	118 (82.5)	49 (74.2)	0.35		V_(df = 2)_ = 0.09
Girls	7 (17.5)	25 (17.5)	17 (25.8)			
ADOS-2 CSS	8.0 (± 1.7)^°°^	7.3 (± 1.7)	7.3 (± 2.2)^**^	0.13		eta^2^ = 0.02
**VABS-II standard score**						
Communication	74.4 (± 18.6)	73.7 (± 17.0)	71.7 (± 17.8)	0.80		eta^2^ = 0.00
Socialization	71.9 (± 13.3)	70.2 (± 13.6)	64.9 (± 15.6)	0.13		eta^2^ = 0.03
Daily living skills	75.1 (± 15.8)	72.4 (± 15.5)	72.9 (± 16.1)	0.71		eta^2^ = 0.00
Best estimate intellectual functioning	55.8 (± 22.3)^**^	78.3 (± 30.1)^¤¤^	94.6 (± 24.9)^*^	< 0.001	G1≠ G2≠ G3	eta^2^ = 0.17
**Child education during lockdown**						
Yes	36 (90.0)	132 (92.3)	57 (86.4)	0.36		V_(df = 2)_ = 0.09
No	4 (10.0)	11 (7.7)	9 (13.6)			
**Continuation of specialized care during lockdown**						
Yes	37 (92.5)	135 (94.4)	55 (83.3)	0.04	G2≠ G3	V_(df = 2)_= 0.17
No	3 (7.5)	8 (5.6)	11 (16.7)			
**Leisure activities**						
At least once a week	7 (17.5)	33 (23.2)^*^	12 (18.2)	0.67		V_(df = 4)_= 0.07
Less than once a week	4 (10.0)	13 (9.2)	10 (15.2)			
Never	29 (72.5)	96 (67.6)	44 (66.7)			
**Family characteristics**						
**Parents' SES**						
High/middle	22 (56.4)^*^	82 (58.6)^¤^	38 (59.4)^**^	0.96		V_(df = 2)_= 0.02
Low	17 (43.6)	58 (41.4)	26 (40.6)			
**Single parent family**						
Yes	9 (22.5)	35 (24.5)	20 (30.3)	0.59		V_(df = 2)_= 0.07
No	31 (77.5)	108 (75.5)	46 (69.7)			
**Number of siblings**						
None	15 (37.5)	32 (22.7)^**^	7 (10.8)^*^	0.02	G1≠ G3	V_(df = 4)_= 0.15
1 sibling	14 (35.0)	51 (36.2)	25 (38.5)			
≥ 2 siblings	11 (27.5)	58 (41.1)	33 (50.8)			
**Mother's educational level**						
High school or lower	9 (29.0)^°^	48 (39.0)^$$^	22 (40.0)^°°^	0.55		V_(df = 2)_= 0.08
University	22 (71.0)	75 (61.0)	33 (60.0)			
**Father's educational level**						
High school or lower	13 (41.9)^°^	59 (48.4)^*$*^	24 (46.2)^$^	0.81		V_(df = 2)_= 0.05
University	18 (58.1)	63 (51.6)	28 (53.9)			

Data are given in mean (SD) or n (%).

* 1 missing data;

** 2 missing data;

¤ 3 missing data;

¤¤ 4 missing data;

° 9 missing data;

°° 11 missing data;

$ 14 missing data;

$$ 20 missing data;

$ 21 missing data.

For Chi^2^ and Fisher's Test, corresponding effect size is Cramer's V. Qualitative conventions for V when df = 1: 0.10 = small, 0.30 = medium, 0.50 = large; when df = 2: 0.07 = small, 0.21 = medium, 0.35 = large; when df = 4: 0.05 = small, 0.15 = medium, 0.25 = large.

For ANOVA, corresponding effect size is eta^2^. Qualitative conventions for eta^2^: 0.01 = small, 0.06 = medium, 0.14 = large (62).

ADOS-2 CSS, autism diagnostic observation schedule second version calibrate severity scale; VABS-II, Vineland second version; SES, socioeconomic status.

### Screen time in ASD children and adolescents during lockdown

Excessive screen time was found in 37.4% (*n* = 93) of our sample and was significantly related to age [*p* < 0.001, Cramer's V_(df = 2)_ = 0.33] as follows: 63.6%, ≥12 year (*n* = 42); 27.3%, 6–11 year (*n* = 39) groups; and 30%, <6 years group (*n* = 12). Gender was linked to excessive screen time for the ≥12 years group [*p* = 0.03, Cramer's V_(df = 1)_= −0.28], with males more likely to have excessive screen time (83.3%) than females (16.7%). Highest screen times (>4 h/day) were: 16.7%, <6 year; 20.5%, aged 6–11 year; and 50.0%, ≥12 year groups. Almost half of parents (48.8%) reported that their child spent more time using screen since COVID-19 pandemic beginning as 44.0 % reported no change and only 4.0 % reported a screen time decrease.

### Univariate analysis of excessive screen time during lockdown

*Children (*<*12 years)* with excessive screen time vs. within recommended level had significantly: lower VABS-II scores for Daily Living Skills [mean = 69.5 (*SD* = 14.6) vs. 74.28 (*SD* = 15.8), respectively; *p* = 0.02, Cohen's *d* = 0.31], higher CBCL scores for withdrawn [70.7% with borderline or clinical range vs. 53.9%, respectively; *p* = 0.06, Cramer's V_(df = 1)_ = −0.15] and lower parental SES [58.0 vs. 35.7%, respectively; *p* = 0.01, Cramer's V_(df = 1)_ = 0.20].

*Adolescents (*≥*12 years)* with excessive screen time vs. within recommended level had: older age [mean = 14.7 years (*SD* = 1.5) vs. 13.4 years (*SD* = 1.9), respectively; *p* = 0.002, Cohen's *d* = −0.76]; male gender (83.3 vs. 58.3%, respectively; *p* = 0.03, Cramer's V_(df = 1)_ = −0.28]; higher CSS [mean = 7.7 (*SD* = 2.2) vs. 6.7 (*SD* = 2.01), respectively; *p* = 0.04, Cohen's *d* = −0.45] and CBCL score for withdrawn (65.7% with borderline or clinical range vs. 35.3% respectively; *p* = 0.04, Cramer's V_(df = 1)_= −0.29]. In addition, they received less specialized care services during lockdown [76.2 vs. 95.8%, respectively, *p* = 0.05, Cramer's V_(df = 1)_= −0.25] and had lower maternal education level [*p* = 0.09, Cramer's V_(df = 1)_= −0.23].

### Multivariable assessment of risk for excessive screen time during lockdown

*Children (*<*12 years):* after adjustment, higher withdrawn CBCL score (borderline/clinical range) and low parental SES were both noted to increase risk of excessive screen time: (ORa = 2.39 (95%CI 1.06–5.36, *p* = 0.04); and ORa = 2.67 (95%CI 1.25–5.71, *p* = 0.01]), respectively. Using multiple imputation of missing data, the sensitivity analysis confirms the result of the complete case multivariable analysis in children (Table 2).

**Table 2:** Risk factors for excessive screen time in children bellow 12 years old.

	**Unadjusted OR**			**Complete case multivariable logistic regression** ^*^ **(41 cases/104 controls)**		**Multivariable logistic regression with multiple imputations** ^*^ **(51 cases/132 controls)**	
	***N***	**OR (95%CI)**	***P***	**aOR (95%CI)**	***P***	**aOR (95%CI)**	***P***
**VABS-II communication SS** (units=10)	183	0.85 (0.70–1.02)	0.08				
**VABS-II daily living skills SS** (units=10)	183	0.82 (0.66–1.01)	0.07				
**VABS-II socialization SS** (units=10)	183	0.85 (0.67–1.08)	0.19				
**CBCL aggressive behavior T-score**							
Borderline/Clinical range vs. Normal range	145	2.03 (0.97–4.28)	0.06				
**CBCL attention problems T-score**							
Borderline/Clinical range vs. Normal range	145	1.65 (0.78–3.51)	0.19				
**CBCL Withdrawn T-score**							
Borderline/Clinical range vs. Normal range	145	2.07 (0.95–4.50)	0.07	2.39 (1.06–5.36)	0.04	2.19 (1.01–4.75)	0.04
**CBCL Anxious/depressed T-score**							
Borderline/Clinical range vs. Normal range	145	1.93 (0.87–4.30)	0.11				
**Parents' SES**							
Low vs. Middle/High	179	2.49 (1.28–4.86)	0.01	2.67 (1.25–5.71)	0.01	2.71 (1.36–5.40)	0.01

* Adjusted on the delay between the date of the last CBCL and the date of the COVID-19 questionnaire.

VABS-II, Vineland second version; SS, standard score; CBCL, Child Behavior Checklist; SES, socioeconomic status.

*Adolescents (*≥*12 years):* after backward selection, only older age was a risk factor of excessive screen time [OR = 2.24 (1.30–3.86), *p* = 0.04]. The sensitivity analysis conducted on imputed data showed that child age and male gender were significant risk factors for excessive screen time in adolescents: ORa = 1.64 (95%CI: 1.53–2.34; *p* = 0.01) and ORa = 4.25 (95%CI: 1.67–15.47; *p* = 0.03), respectively (Table 3).

**Table 3:** Risk factors for excessive screen time in adolescents aged 12 years old and over.

	**Unadjusted OR**			**Complete case multivariable logistic regression** **(31 cases/16 controls)**		**Multivariable logistic regression with multiple imputation** **(42 cases/24 controls)**	
	***N***	**OR (95%CI)**	***P***	**aOR (95%CI)**	***P***	**aOR (95%CI)**	***P***
**Child's age** (years)	66	1.61 (1.14–2.28)	0.01	2.24 (1.30–3.86)	0.004	1.64 (1.53–2.34)	0.01
**Gender**: Boy vs. Girl	66	3.57 (1.13–11.25)	0.03			4.25 (1.67–15.47)	0.03
**ADOS-2 CSS**	64	1.23 (0.96–1.57)	0.10				
**Father's education**							
Elementary/High school vs. College/University	52	2.60 (0.80–8.51)	0.11				
**Mother's education**							
Elementary/High school vs. College/University	52	2.83 (0.85–9.50)	0.09				
**Continuation of special care during containment**							
No vs. Yes	66	7.19 (0.86–60.14)	0.07				
**CBCL Withdrawn T-score**							
Normal range vs. Borderline/Clinical range	52	3.51 (1.04–11.85)	0.04				

ADOS-2 CSS, autism diagnostic observation schedule second version calibrate severity scale; VABS-II, Vineland second version; SS, standard score; CBCL, Child Behavior Checklist.

## Discussion

The current study provided a unique opportunity to examine screen time during a discrete COVID-19 lockdown period among a sample of 249 children and adolescents with well-characterized diagnosis of ASD enrolled in a regional ASD cohort in France. We found that 37.4% of subjects had above recommended levels of screen time during lockdown. This figure is comparable to that obtained in the U.S. general population in the National Health and Nutrition Examination Survey that reported sedentary behavior profiles comprised of television viewing or engaging with electronic media that exceeded 2-h/day or more of screen time for 47% of the population of 2–15 year old children and adolescents (27). It is notable that the latter study classified sedentary behaviors depending on the screen time activities involved as “productive” (e.g., computer use for homework or reading, or other educational activity) or “leisure” (e.g., viewing television or playing computer or video games).

Regarding the impact of lockdown on children's screen time, half of parents reported an increase since the beginning of the pandemic, which is congruent with previous studies (39). This indicates that screen time measured during the pandemic does not necessarily represent the usual screen time of children. In particular, children and adolescents had to adapt to a new virtual system for education and work purposes, so that “excessive” screen time can be biased by this constrains during the lock-down.

The results of the current study were consistent with that of Must et al. (32) that compared children with ASD and typically developing children under 12 years of age. Must et al. (32) reported significantly elevated screen times, 2.5 vs. 1.6 h/day, among ASD and non-ASD children, respectively, albeit during a non-lockdown period. A study by Krupa et al. (30) involving 2–4 year-old children with ASD vs. typically developing non-ASD children also reported significantly elevated weekly screen times, 8.4 vs. 6.9 h, respectively, that did not exceed recommended levels. Nonetheless, although not at excessive screen time levels, for these young children, the Krupa et al. (30) study emphasized the importance of joint family-child screen time that had an impact on mother-child reciprocal interaction.

The present study allowed to identify factors associated to excessive screen time. First, above recommended screen time was higher for adolescents (63%), almost double that of under 12-year-olds. Excessive screen time experience among adolescents is also supported by prior studies and likely relate to ease of screen access, attraction to social networks, and difficulty in implementing stricter controls (19, 63). Second, our analysis found that children with excessive screen time had lower daily living skills (as assessed with VABS-II). This is consistent with the previously reported association of excessive screen time with negative health conditions in children and adolescents with ASD (29, 33, 37). Third, children and adolescents with ASD with excessive screen time were significantly more withdrawn (as assessed with CBCL) suggesting that children with these clinical characteristics may are more likely to have greater screen time. However, it is difficult to understand the true nature of this interaction, which can reflect either greater interest in solitary screen-based activities, or adverse effect of excessive screen time on prosocial behavior. Alternatively, the degree of withdrawal observed in children and adolescents with excessive screen time could be related to greater autism severity, as noted by Dong et al. (64), who found that children with significant sensory characteristics, were more likely to be fascinated by the visual stimuli of screens. It is also possible that they were given these devices because it was a convenient way to keep them occupied during the lockdown. Fourth, the univariate analysis found that adolescents with excessive screen time were not only older but were more likely to be males, a finding again consistent with previous literature (19, 63). Fifth, the adolescents with excessive screen time received less special care services and less educational resources at home. In such uniformed circumstances, it has also been noted that parents themselves may be involved in excessive screen time (65). As already been reported in previous studies (19) low parental SES has also been noted to be a risk factor for excessive screen time among children with less opportunities for non-screen based leisure and vocational activities.

Finally, the multivariable and sensitivity analyses, underscored that be more withdrawn and had lower parental SES were the risk factors for excessive screen time among children. Among adolescents, older age and male gender were the risk factors for excessive screen time, two factors commonly supported by prior population based studies (17–20, 63). Number of limitations of the study should be noted. The subsample of children and adolescents examined for the present study were younger and had higher intellectual and adaptive functioning scores than in the overall ELENA cohort, which limits the generalization of our results. ELENA is a regional cohort and not a nationally representative sample, which may bias our results on the link between socio-economic status and screen time and therefore we cannot generalize. Data on socio-familial characteristics were only available for a subset of children, who did not differ from the whole cohort on these variables. We do not have a measurement of the screen exposure before confinement or a comparison to the population without ASD or the general population. Part of the clinical data used in this article was collected at the last visit in ELENA about 12 months before our COVID-19 survey. However, the clinical presentation in other cohorts with ASD (66) was found to be stable in the short term. Nevertheless, an important strength of the current study included the use of a large sample of children and adolescents with confirmed ASD diagnosis with consideration of a wide range of clinical and socio-familial variables collected using validated instruments and potential risk factors. Although the sample size was limited, the significance of the findings for a relatively small subgroup was important and consistent with prior research. Given the positive relationship found between age and excessive screen time, future research is needed to examine long-term effects of excessive screen time from adolescence onwards.

### Implications

The experience of above recommended level of screen time among children and adolescents with ASD is equally important with consequent negative effects of excessive screen time on health. It is therefore paramount to develop recommendations on screen time and to provide guidance to parents of children and adolescents with ASD. Further studies are needed to examine the relationship between sedentary behaviors, screen time and healthy and behavioral outcomes in children and adolescents with ASD. The clinical and social risk factors identified in the current study also make it possible for professionals to consider measures to implement the more optimal use of screen time activities for educational, therapeutic and social goals. It is particularly important to encourage the access of children to non-screen time based leisure and cultural activities that complement educational interventions in particular during period of lockdowns.

## Data availability statement

The datasets presented in this article are not readily available because Research data are not shared due to the need for confidentiality. The corresponding author, AB, confirms that she had full access to all the data in the study and takes responsibility for the integrity of the data and the accuracy of the data analysis. Requests to access the datasets should be directed to AB, rech-clinique-autisme@chu-montpellier.fr.

## Ethics statement

This study involving human participants was reviewed and approved by Internal Review Board of the University Hospital of Montpellier. Written informed consent to participate in this study was provided by the participants' legal guardian/next of kin.

## Informed consent

Signed informed consent is obtained from all participating families included in the ELENA cohort.

## ELENA study group

Amaria Baghdadli, Catherine Chabaux, Clarisse Chatel, David Cohen, Emmanuel Damville, Marie-Maude Geoffray, Ludovic Gicquel, Renaud Jardri, Thierry Maffre, Alexandre Novo, Roxane Odoyer, Marie-Joëlle Oreve, Didier Périsse, François Poinso, Julien Pottelette, Laurence Robel, Catherine Rolland, Marie Schoenberger, Sandrine Sonié, Mario Speranza, and Stéphanie Vespérini.

## Author contributions

MB and AB conceived the study, contributed to the collection, analysis, interpretation of the data, and drafted the manuscript. AB is the PI of the ELENA cohort. JL contributed to the collection of the data. MP and M-CP analyzed and interpreted the data and critically revised it for the principal intellectual content. All authors reviewed and approved the final version.

## Funding

This work was supported by the French Health Ministry (DGOS) PHRCN 2013 [Grant No 1: 13-0232] and the Caisse Nationale de Solidarité pour l'Autonomie (CNSA) [Grant No 2: 030319]. The CHU of Montpellier (AOI) provided additional support. The funders had no role in study design, data collection and analysis, decision to publish, or preparation of the manuscript.

## Conflict of interest

The authors declare that the research was conducted in the absence of any commercial or financial relationships that could be construed as a potential conflict of interest.

## Publisher's note

All claims expressed in this article are solely those of the authors and do not necessarily represent those of their affiliated organizations, or those of the publisher, the editors and the reviewers. Any product that may be evaluated in this article, or claim that may be made by its manufacturer, is not guaranteed or endorsed by the publisher.
