# Supplementary material for: Screen time and associated risks in children and adolescents with autism spectrum disorders during a discrete COVID-19 lockdown period
Source: Front Psychiatry. 2022 Dec 1;13:1026191. doi: 10.3389/fpsyt.2022.1026191 (PMC9751585; doi:10.3389/fpsyt.2022.1026191)
Supplement: Supplementary file 1 [file Table_1.DOCX]

**Supplementary Table S1.**  Comparison of clinical characteristics between participants included in the analyses and the other participants involved in the ELENA Cohort

|  | **Participants included in our study**  **N=249** | **Other participants included in ELENA Cohort**  **N=638** | **Pvalue** | **Effect size** |
| --- | --- | --- | --- | --- |
| **Children and adolescents’ characteristics** | |  |  |  |
| Gender  Boys  Girls | 200 (80.30%)  49 (19.70%) | 534 (83.7%)  104 (16.3%) | 0.23 | V_(df=1)_= 0.04 |
| Age during quarantine (years) | 9.12 (±3.75) | 9.83 (±4.09) | 0.04 | d= 0.18 |
| Best estimate intellectual functioning | 79.11 (±30.18)* | 72.17 (±29.64)^¤^ | 0.002 | d= -0.23 |
| ADOS-2 CSS | 7.37 (±1.83)** | 7.31 (±1.92)^££^ | 0.72 | d= -0.03 |
| VABS II standard score  Communication  Daily living skills  Socialization | 73.27 (±17.44)  72.95 (±15.70)  69.08 (±14.28) | 68.11 (±17.58)°  69.47 (±14.59)°  67.10 (±14.66)°° | <0.001  0.003  0.05 | d= -0.29  d= -0.23  d= -0.14 |
| **Family characteristics** |  |  |  |  |
| Mother’s age (years) | 40.70 (±6.32)°° | 41.04 (±6.64)^&&^ | 0.50 | d= 0.05 |
| Father’s age (years) | 43.98 (±7.30)^$^ | 44.16 (±7.59)^¥^ | 0.98 | d= 0.02 |
| Mother’s educational level  High school or lower  University | 79 (37.8%)^¤¤^  130 (62.2%) | 136 (40.0%)^¥¥^  204 (60.0%) | 0.61 | V_(df=1)_= 0.02 |
| Father’s educational level  High school or lower  University | 96 (46.8%)^£^  109 (53.2%) | 166 (49.7%)^§^  168 (50.3%) | 0.52 | V_(df=1)_= 0.03 |
| Parents’ SES  High/middle  Low | 142 (58.4%)^$$^  101 (41.6%) | 238 (56.5%)^&^  183 (43.5%) | 0.63 | V_(df=1)_= -0.02 |

Data are given in mean (SD) or n (%).

° 2 missing value, °° 3 missing values, ^$^ 4 missing values, ^$$^ 6 missing values, * 7 missing values, **13 missing data, ^¤^ 27 missing data, ^¤¤^ 40 missing data,  ^£^ 44 missing data, ^££^ 45 missing data, ^&^217 missing data, ^&&^223 missing data, ^¥^230 missing data, ^¥¥^298 missing data, ^§^334 missing data.

For Chi², corresponding effect size is Cramer’s V. Qualitative conventions for V when df=1: 0.10 = small, 0.30 = medium, 0.50 = large. For T test, corresponding effect size is Cohen’s d. Qualitative conventions for d: 0.20 = small, 0.50 = medium, 0.80 = large (Cohen, 1992).

ADOS-2 CSS, autism diagnostic observation schedule second version calibrate severity scale; VABS-II, Vineland second version; SES, socioeconomic status.
